# Supplementary material for: Formal [3+2] Cycloaddition Reactions of Electron-Rich Aryl Epoxides with Alkenes under Lewis Acid Catalysis Affording Tetrasubstituted Tetrahydrofurans
Source: Molecules. 2020 Feb 6;25(3):692. doi: 10.3390/molecules25030692 (PMC7036929; doi:10.3390/molecules25030692)
Supplement: Supplementary file 1 [file molecules-25-00692-s001.pdf]

Supporting Information

Formal [3+2] Cycloaddition Reactions of Epoxides with Alkenes under Lewis Acid Catalysis Affording Tetrasubstituted Tetrahydrofurans

Víctor E. Macías,[‡] Luís Cuca-Suárez,[‡] Santiago Rodríguez,[§] and Florenci V. González*[,§]

[§] Departament de Química Inorgànica i Orgànica, Universitat Jaume I. Castelló, Spain.

[‡] Departamento de Química, Universidad Nacional de Colombia. Bogotá D.C., Colombia.

Contents

General Information……………………………………………………………………..S3

Experimental procedures ……………………………….……………………………...S3

Spectroscopic data of compounds……………………..…………………………..S3-S5

NMR Spectra of compounds……………….………………………………...…S6-S14

General information:
Unless otherwise specified, all reactions were carried out under nitrogen atmosphere with magnetic stirring. All solvents and reagents were obtained from commercial sources and were purified according to standard procedures before use. ^{1}H NMR spectra and ^{13}C NMR spectra were measured in $CDCl_3$ (^{1}H, 7.24 ppm; ^{13}C 77.0 ppm) solution at 30 °C on a 300 MHz or a 500 MHz NMR spectrometer. Mass spectra were measured in a QTOF I (quadrupole-hexapole-TOF) mass spectrometer with an orthogonal Z-spray-electrospray interface. EM Science Silica Gel 60 was used for column chromatography while TLC was performed with precoated plates (Kieselgel 60, F_{254}, 0.25 mm).

Preparation of epoxides 2-(3,4-dimethoxyphenyl)-3-methyloxirane and 2,3-diphenyloxirane. A stirrred solution of the alkene (5.61 mmol) in dichloromethane (20 mL) cold with an ice-bath was treated with sodium carbonate (10 % aqueous solution) (40 mL). Then a solution of metachloroperbenzoic acid (12.4 mmol) in dichloromethane (20 mL) was added dropwise to the previous resulting mixture cold with an ice-bath. The resulting mixture was stirred for 20 minutes. Then was poured into a separatory funnel. Organic layer was separated and washed sequentially with sodium carbonate (10 % aqueous solution) (5 x 25 mL) and with and dried over Na_2SO_4. Then the solvent was evaporated and the residue was purified by column chromatography (silicagel, n-hexane / ethyl acetate; 9:1).

Preparation of methyl 3-(3,4-dimethoxyphenyl)oxirane-2-carboxylate.
Sodium (62 g) was added to dry methanol (900 mL) and the resulting solution was cold to -10°C. Then a solution of veratraldehyde (1.8 mol) and methyl chloroacetate (293 g) in methanol (mL) was added dropwise for a period of 3 h with vigorous stirring. Then resulting mixture was stirred for 2 h at -5°C, and then 3 h at room temperature. Then the mixture was poured into a flask containing a mixture of water, ice and acetic acid (20 mL) (total volume of the mixture was 3.5 L). Desired compound precipitated as a white powder, then was filtered and washed with cold water, and dried. The solid was recrystallized from methanol to afford a white solid (65-66 ºC).

General experimental procedure for the preparation of tetrahydrofurans.
To an ice-bath cold solution of alkene (0.56 mmol) in dichloromethane (1 mL) was added the corresponding epoxide (0.56 mmol, 1 equiv), and then aluminum chloride (0.168 mmol, 0.3 equiv). The resulting mixture was stirred cold with an ice-bath for 30 minutes. An indicating color change was observed after this time. Then water (10 mL) was added and extracted with dichloromethane (3 x 20 mL). Combined organic layers were dried (Na_2SO_4), filtered and concentrated to afford an oil which was purified through silica gel column.

(2*R*,3*R*,4*S*,5*R*)-2,4-bis(3,4-dimethoxyphenyl)-3,5-dimethyltetrahydrofuran 1a and (2*R*,3*R*,4*S*,5*S*)-2,4-bis(3,4-dimethoxyphenyl)-3,5-dimethyltetrahydrofuran 2a. (156 mg, 75%) (hexanes/ethyl acetate, 75/25 and 60/40). **1a**: ^{1}H-NMR (300 MHz, $CDCl_3$) d 7.00 (m, 2H), 6.86 (m, 2H), 6.72 (m, 2H), 4.46 (m, 1H), 3.92 (s, 3H), 3.87 (s, 3H), 3.18 (dd, J = 9.6, 8.5 Hz, 1H), 2.32 (m, 1H), 1.05 (d, J = 6.5 Hz, 3H), 0.97 (d, J = 6.5 Hz, 3H) ppm; ^{13}C-NMR (75 MHz, $CDCl_3$) d 149.2, 149.0, 148.8, 148.0, 134.3, 132.2, 120.8, 118.7, 112.4, 111.3, 111.2, 109.8, 87.7, 77.6, 77.4, 56.8, 56.2, 56.1, 56.0, 46.8, 19.1, 15.3 ppm. HRMS (ESI) calcd for $C_{22}H_{28}O_2Na$ (M + Na^+) 395.1834, found 395.1831.). **2a**: ^{1}H NMR (300 MHz, $CDCl_3$) δ 6.89 (m, 2H), 6.76 (m, 2H), 6.64 (m, 2H), 4.49 (d, *J* = 9.4 Hz, 1H), 4.22 (dq, *J* = 12.0, 6.0 Hz, 1H), 3.85 (s, 3H), 3.81 (s, 3H), 3.80 (s, 3H), 3.79 (s, 3H), 2.46 (dd, J = 11.0, 9.5 Hz, 1H), 2.32 (m, 1H), 1.22 (d, *J* = 6.0 Hz, 3H), 0.86 (d, *J* = 6.5 Hz, 5H) ppm.

(2*R*,3*S*,4*R*,5*R*)-3-(3,4-dimethoxyphenyl)-2,4-dimethyl-5-phenyltetrahydrofuran 1b and (2*R*,3*S*,4*R*,5*S*)-3-(3,4-dimethoxyphenyl)-2,4-dimethyl-5-phenyltetrahydrofuran 2b. (108 mg, 62 %) (hexanes/ethyl acetate, 75/25 and 60/40). ^{1}H-NMR (300 MHz, $CDCl_3$) d 7.29 – 7.45 (m, 5H), 6.68 – 6.82 (m, 3H), 4.46 (m, 1H), 4.49 (d, J = 9.1 Hz, 1H), 3.86 (s, 3H), 3.84 (s, 3H), 3.18 (dd, J = 9.6, 8.2 Hz, 1H), 2.31 (m, 1H), 1.05 (d, J = 6.5 Hz, 3H), 0.97 (d, J = 6.5 Hz, 3H) ppm; ^{13}C-NMR (100MHz, $CDCl_3$) d 148.8, 147.5, 141.5, 131.9, 128.2, 127.6, 126.0, 120.6, 111.9, 111.0, 87.5, 56.6, 55.7, 47.0, 18.8, 15.0 ppm. HRMS (ESI) calcd for $C_{20}H_{24}O_3Na$ (M + Na^+) 335.1623, found 335.1625. **2b**: ^{1}H-NMR (400 MHz, $CDCl_3$) d 7.39 – 7.20 (m, 5H), 6.62 – 6.77 (m, 3H), 4.54 (d, J = 9.4 Hz, 1H), 4.29 - 4.17 (m, 1H), 3.80 (s, 3H), 3.79 (s, 3H), 2.47 (dd, J = 11.1, 9.5 Hz, 1H), 2.18 (m, 1H), 1.22 (d, J = 6.0 Hz, 3H), 0.87 (d, J = 6.5 Hz, 3H) ppm.

(2*S*,3*R*,4*S*,5*R*)-2-(3,4-dimethoxyphenyl)-3-methyl-4,5-diphenyltetrahydrofuran 1c. (31 mg, 15 %) (hexanes/ethyl acetate, 75/25 and 60/40). ^{1}H-NMR (500 MHz, $CDCl_3$) d 6.56 – 6.79 (m, 6H), 6.90 – 6.93 (m, 2H), 7.19 – 7.33 (m, 5H), 4.04 (d, J = 9.6 Hz,1H), 3.88 (d, J = 9.2 Hz, 1H), 3.82 (s, 3H), 3.70 – 3.79 (m, 1H), 3.75 (s, 3H), 2.13 (m, 1H), 0.96 (d, J = 6.4 Hz, 3H) ppm; ^{13}C-NMR (100 MHz, $CDCl_3$) d 149.1, 147.7, 143.2, 139.9, 138.5, 137.5, 129.9, 129.3, 128.9, 127.2, 126.2, 126.1, 122.1, 112.2, 111.0, 78.0, 77.2, 56.0, 55.9, 53.7, 43.3, 16.0 ppm. HRMS (ESI) calcd for $C_{20}H_{24}O_3Na$ (M + Na^+) 397.1780, found 397.1779.

(2*R*,3*S*,5*R*)-3-(3,4-dimethoxyphenyl)-2-methyl-5-phenyltetrahydrofuran 1d. (95 mg, 57 %) (hexanes/ethyl acetate, 75/25 and 70/30). ^{1}H NMR (300 MHz, $CDCl_3$) δ 7.63 – 7.17 (m, 8H), 6.96 – 6.62 (m, 5H), 5.23 (dd, *J* = 12.0, 5.6 Hz, 1H), 5.07 (dd, *J* = 9.5, 6.7 Hz, 1H), 4.52 – 4.39 (m, 1H), 4.24 (dq, *J* = 9.1, 6.0 Hz, 1H), 4.12 (dq, *J* = 8.8, 6.0 Hz, 1H), 3.62 (dd, *J* = 15.8, 7.8 Hz, 1H), 3.06 (ddd, *J* = 11.6, 9.2, 6.8 Hz, 1H), 2.94 (q, *J* = 8.9 Hz, 1H), 2.78 (dd, *J* = 12.5, 6.5 Hz, 1H), 2.74 (dd, *J* = 12.6, 2.2 Hz, 1H), 2.60 (t, *J* = 8.7 Hz, 1H), 2.56 (t, *J* = 8.7 Hz, 1H), 2.40 (dd, *J* = 8.8, 5.5 Hz, 1H), 2.35 (dd, *J* = 8.8, 5.4 Hz, 1H), 2.18 (dt, *J* = 22.1, 11.1 Hz, 1H), 2.25 (m, 1H), 1.40 (d, *J* = 6.0 Hz, 2H), 1.36 (d, *J* = 6.0 Hz, 1H), 1.00 (d, *J* = 6.4 Hz, 1H); ^{13}C-NMR (75 MHz, $CDCl_3$) d 149.1, 149.1, 148.7, 148.0, 147.9, 147.6, 143.8, 143.6, 142.7, 128.5, 128.4, 128.4, 125.8, 125.7, 125.6, 119.6, 119.5, 111.7, 111.4, 111.4, 110.9, 110.8, 110.7, 82.8, 82.3, 80.1, 79.9, 79.8, 78.5, 55.9, 55.9, 55.8, 54.1, 51.8, 48.6, 44.7, 43.8, 41.1, 19.6, 19.1, 18.0 ppm. HRMS (ESI) calcd for $C_{20}H_{24}O_3Na$ (M + Na^+) 321.1468, found 321.1468.

(2*R*,3*S*,4*R*,5*R*)-3-(3,4-dimethoxyphenyl)-2,4-dimethyl-5-(2,4,5 trimethoxyphenyl)tetrahydrofuran 1e. (92 mg, 41 %) (hexanes/ethyl acetate, 75/25 and 60/40). ^{1}H-NMR (300 MHz, $CDCl_3$) d 7.07 (s, 1H), 6.73 (s, 1H), 6.62 – 6.67 (m, 2H), 6.46 (s, 1H), 4.86 (d, J = 9.0 Hz,1H), 4.36 (dq, J = 8.2, 6.5 Hz, 1H), 3.82 (s, 3H), 3.80 (s, 3H), 3.79 (s, 3H), 3.78 (s, 3H), 3.73 (s, 3H), 3.09 (dd, J = 9.1, 8.4 Hz, 1H), 2.31 (m, 1H), 0.98 (d, J = 6.6 Hz, 3H), 0.89 (d, J = 6.5 Hz, 3H) ppm; ^{13}C-NMR (75.5 MHz, $CDCl_3$) d 151.8, 149.0, 148.8, 147.7, 143.4, 132.4, 97.7, 81.0, 77.2, 56.9, 56.7, 56.5, 56.2, 55.9, 55.9, 46.5, 18.8, 15.4 ppm. HRMS (ESI) calcd for $C_{23}H_{30}O_6Na$ (M + Na^+) 425.1940, found 425.1939.

4-((2*R*,3*R*,5*R*)-4-(3,4-dimethoxyphenyl)-3,5-dimethyltetrahydrofuran-2-yl)-2-methoxyphenol 1f and **4-((2*R*,3*R*,5*S*)-4-(3,4-dimethoxyphenyl)-3,5-dimethyltetrahydrofuran-2-yl)-2-methoxyphenol 2f**. (70 mg, 35 %) (hexanes/ethyl acetate, 75/25 and 60/40). **1f**: ^{1}H-NMR (300 MHz, $CDCl_3$) d 6.91 (s, 1H), 6.85 (s, 1H), 6.62 – 6.78 (m, 3H), 5.57 (br s, 1H), 4.36 (dd, J = 8.1, 6.5 Hz, 1H), 4.35 (d, J = 9.2 Hz,1H), 3.84 (s, 3H), 3.80 (s, 3H), 3.79 (s, 3H), 3.10 (dd, J = 9.9, 8.2 Hz, 1H), 2.22 (m, 1H), 0.96 (d, J = 6.5 Hz, 3H), 0.89 (d, J = 6.5 Hz, 3H) ppm; ^{13}C-NMR (75.5 MHz, $CDCl_3$)

d 148.8, 147.8, 146.6, 145.2, 133.4, 132.0, 120.6, 119.3, 114.2, 112.2, 111.1, 108.9, 87.7, 56.5, 55.9, 55.9, 55.9, 46.6, 18.9, 15.1 ppm. HRMS (ESI) calcd for $C_{21}H_{26}O_5Na$ (M + Na^+) 381.1678, found 381.1679. **2f**: ^{1}H-NMR (300 MHz, $CDCl_3$) d 6.89 (s, 1H), 6.84 (s, 1H), 6.62 – 6.78 (m, 3H), 5.57 (br s, 1H), 4.47 (d, J = 9.4 Hz, 1H), 4.2 (dq, J = 9.4, 6.0 Hz,1H), 3.86 (s, 3H), 3.82 (s, 3H), 3.80 (s, 3H), 2.46 (dd, J = 11.1, 9.4 Hz, 1H), 2.22 (m, 1H), 1.22 (d, J = 6.0 Hz, 3H), 0.85 (d, J = 6.5 Hz, 3H) ppm.

Methyl (2*S*,3*S*,4*R*,5*R*)-3,5-bis(3,4-dimethoxyphenyl)-4-methyltetrahydrofuran-2-carboxylate 1g and **methyl (2*R*,3*S*,4*R*,5*R*)-3,5-bis(3,4-dimethoxyphenyl)-4-methyltetrahydrofuran-2-carboxylate 2g**. (105 mg, 45 %) (hexanes/ethyl acetate, 75/25 and 60/40). **1g**: ^{1}H-NMR (300MHz, $CDCl_3$) d 7.55 (d, J = 1.9 Hz, 1H), 7.10 (m, 1H), 6.88 – 6.71 (m, 4H), 4.81 (d, J = 9.4 Hz, 1H), 4.55 (d, J = 9.7 Hz, 1H), 3.97 (s, 3H), 3.90 (s, 3H), 3.87 (s, 3H), 3.86 (s, 3H), 3.84 (s, 3H), 3.43 (dd, J =11.9, 9.4 Hz, 1H), 2.67 – 2.50 (m, 1H), 0.92 (d, J = 6.5 Hz, 3H) ppm; ^{13}C-NMR (75MHz, $CDCl_3$) d 172.6, 149.4, 149.0, 148.5, 133.1, 128.3, 120.4, 119.5, 111.5, 111.2, 110.7, 110.3, 89.2, 80.9, 56.5, 55.8, 51.4, 45.3, 13.0 ppm. HRMS (ESI) calcd for $C_{23}H_{28}O_7Na$ (M + Na^+) 439.1732, found 439.1733. **2g**: ^{1}H-NMR (300 MHz, $CDCl_3$) δ 7.55 (d, J = 1.9 Hz, 1H), 6.92 (m 5H), 4.71 (d, *J* = 9.1 Hz, 1H), 4.68 (d, *J* = 8.2 Hz, 1H), 3.97 (s, 3H), 3.92 (s, 3H), 3.89 (s, 3H), 3.87 (s, 3H), 3.86 (s, 3H), 3.16 (dd, *J* = 10.8, 8.6 Hz, 2H), 2.19 (m, 1H), 0.95 (d, *J* = 6.5 Hz, 3H) ppm.

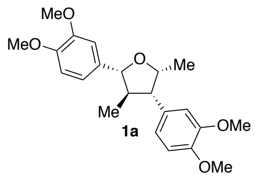
MeO
MeO
O
Me
Me
1a
OMe
OMe

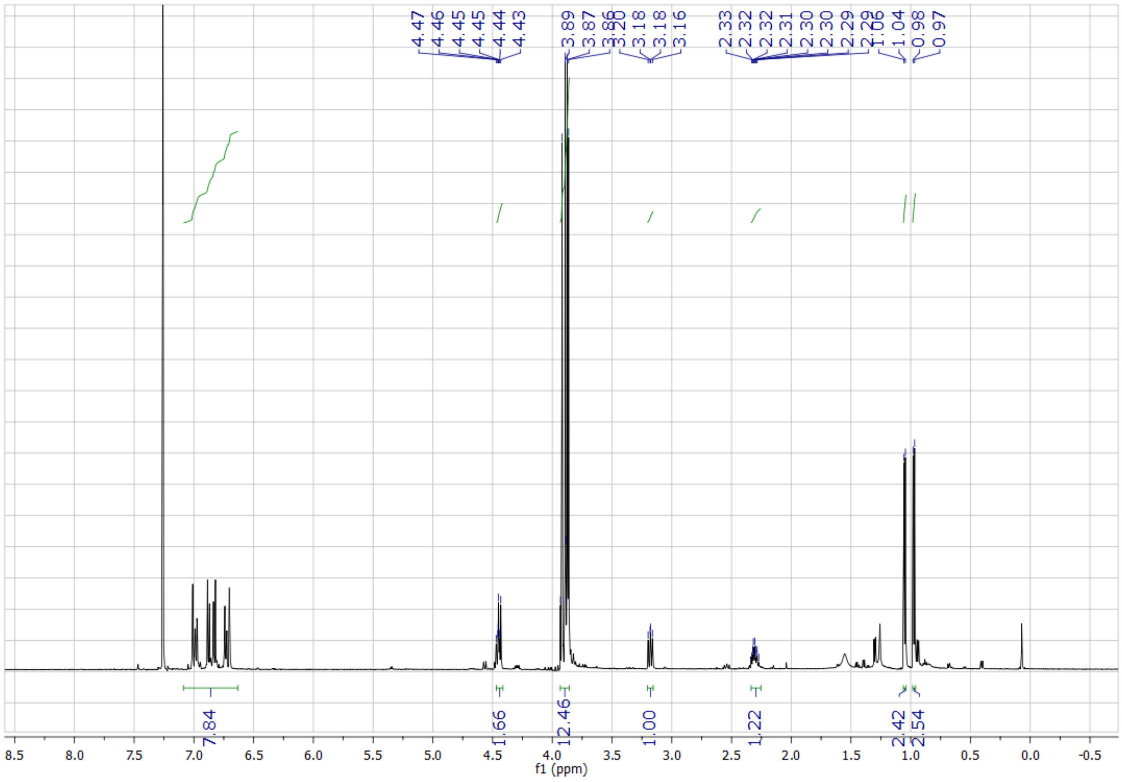
4.47 4.46 4.45 4.45 4.44 4.43
3.89 3.87 3.86 3.20 3.18 3.18 3.16
2.33 2.32 2.32 2.31 2.30 2.30 2.29 2.29 1.06 1.04 0.98 0.97
7.84
1.66
12.46
1.00
1.22
2.42
2.54
8.5 8.0 7.5 7.0 6.5 6.0 5.5 5.0 4.5 4.0 3.5 3.0 2.5 2.0 1.5 1.0 0.5 0.0 -0.5
f1 (ppm)

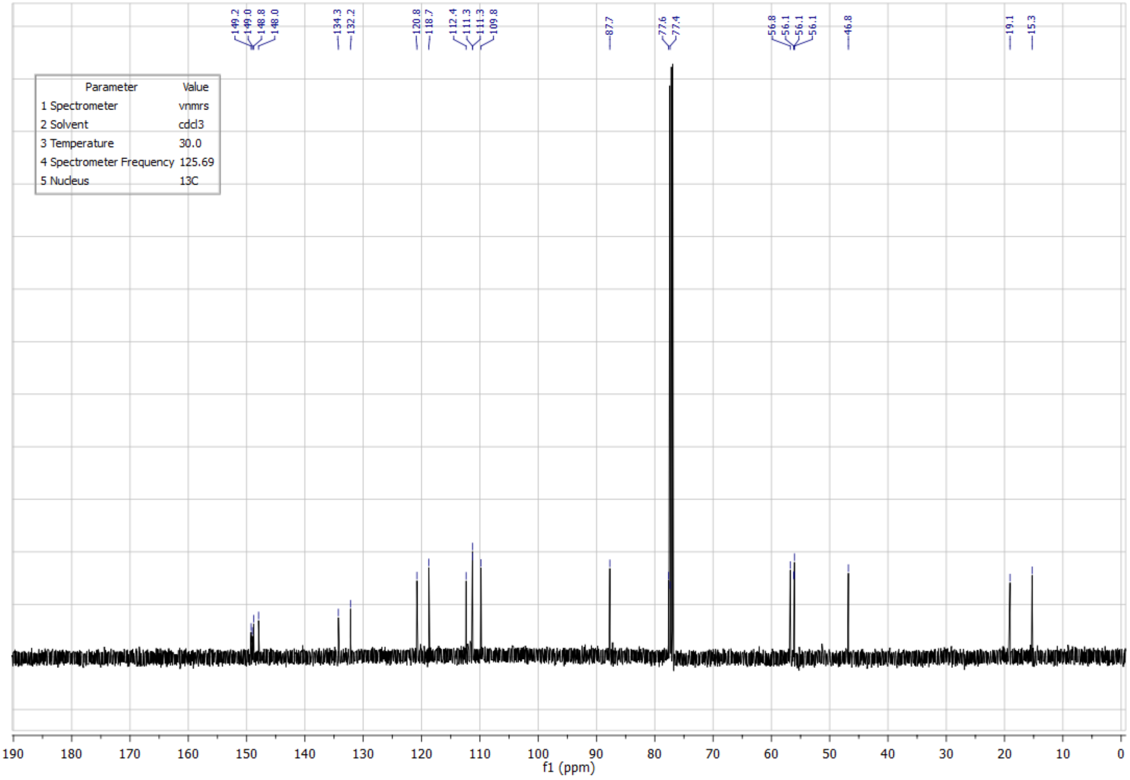
149.2 149.0 148.8 148.0
134.3 132.2
120.8 118.7
112.4 111.3 111.3 109.8
87.7
77.6 77.4
56.8 56.1 56.1 56.1
46.8
19.1
15.3
Parameter Value
1 Spectrometer vnmrs
2 Solvent cdcl3
3 Temperature 30.0
4 Spectrometer Frequency 125.69
5 Nucleus 13C
190 180 170 160 150 140 130 120 110 100 90 80 70 60 50 40 30 20 10 0
f1 (ppm)

COSY **1a**

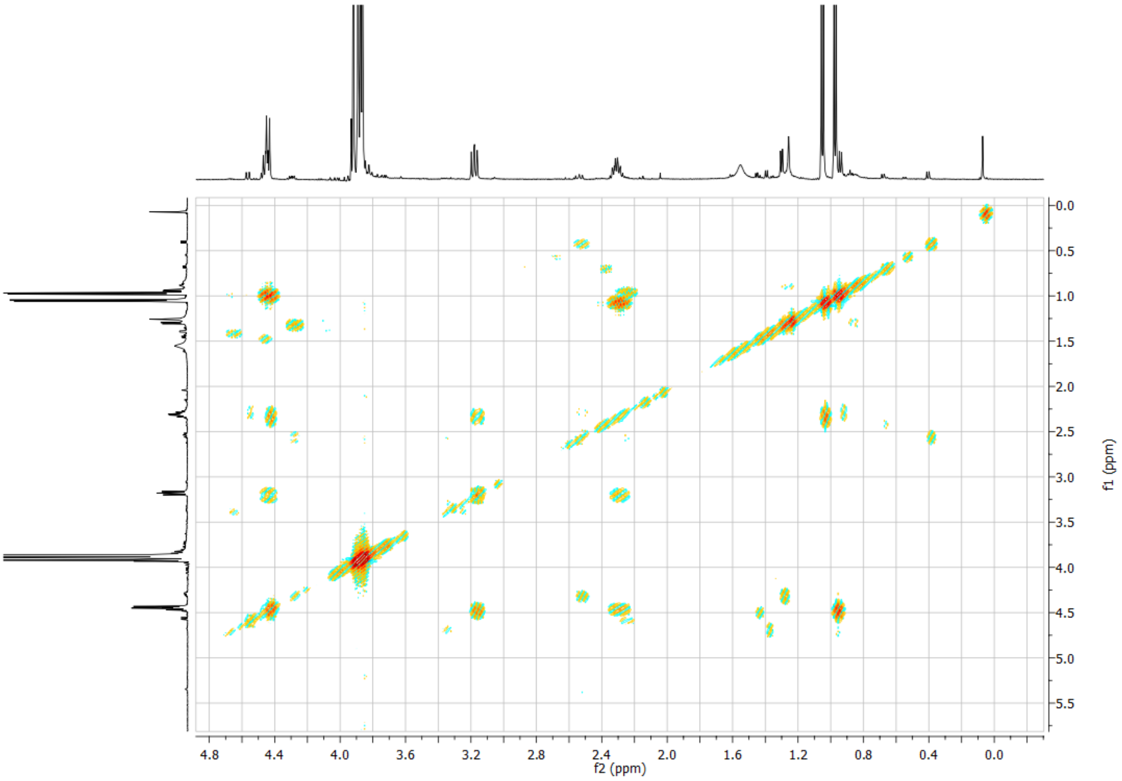

NOESY **1a**

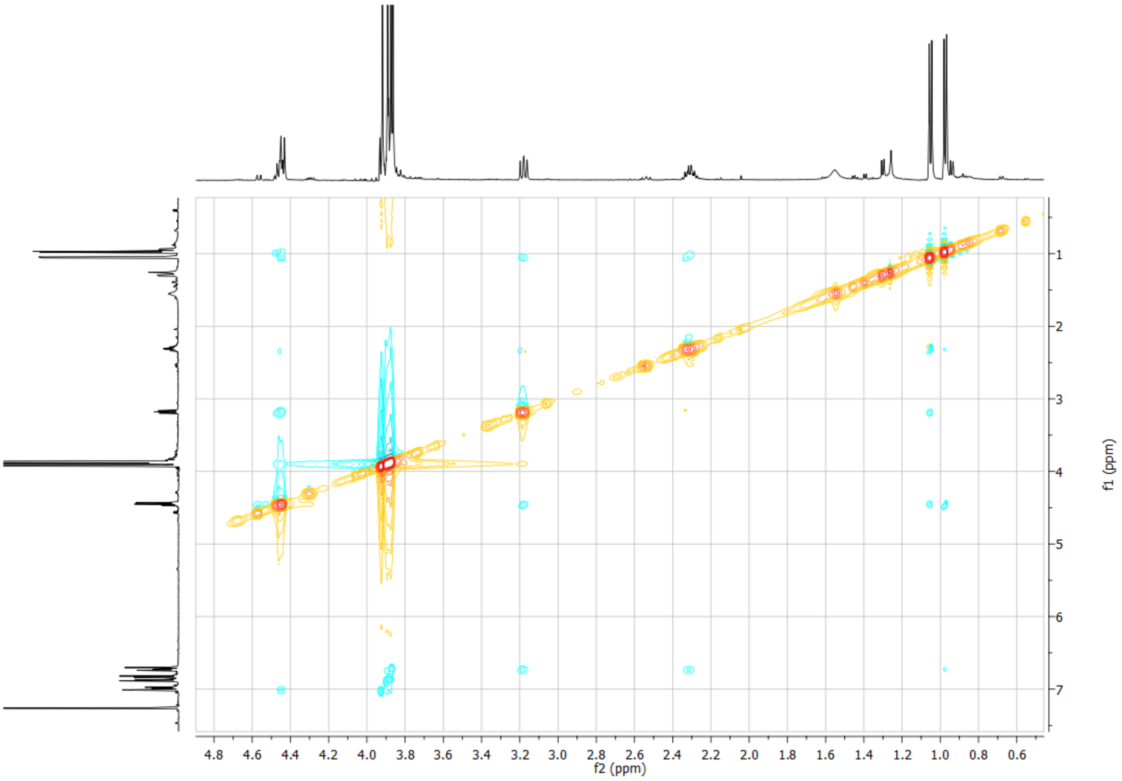

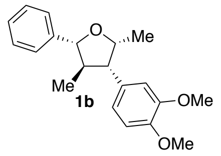
O
Me
Me
OMe
OMe

1b

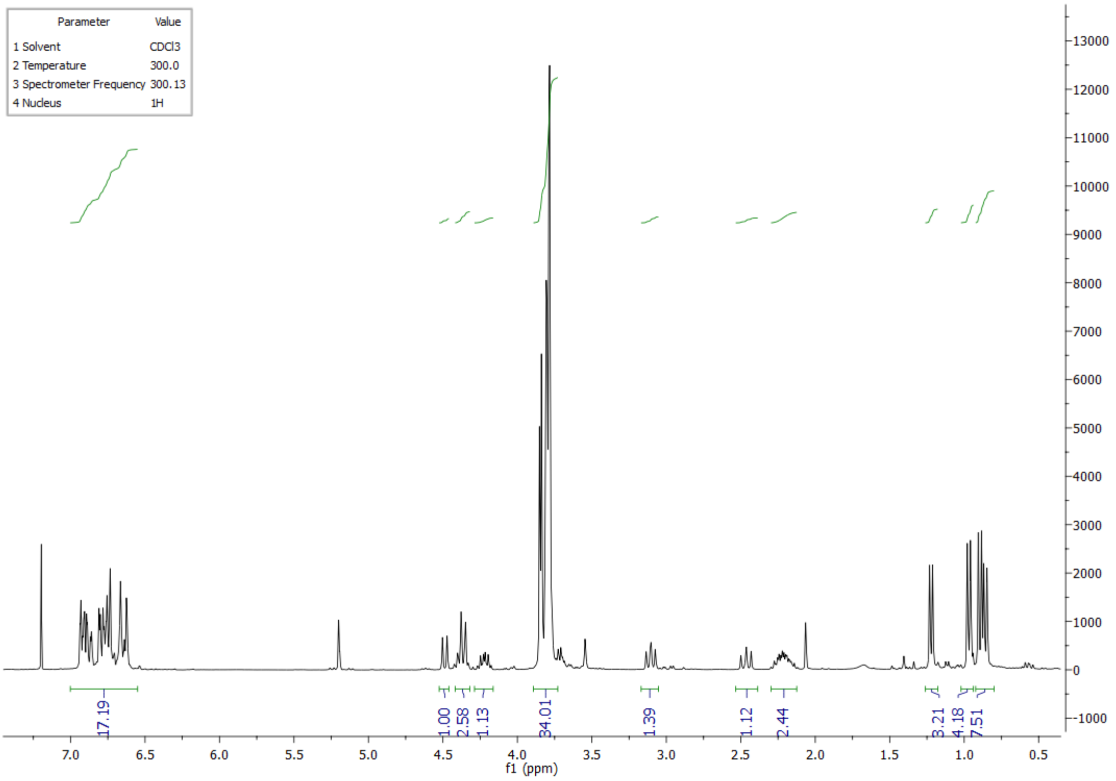
Parameter Value
1 Solvent CDCl3
2 Temperature 300.0
3 Spectrometer Frequency 300.13
4 Nucleus 1H
17.19
1.00
2.58
1.13
34.01
1.39
1.12
2.44
3.21
4.18
7.51
f1 (ppm)

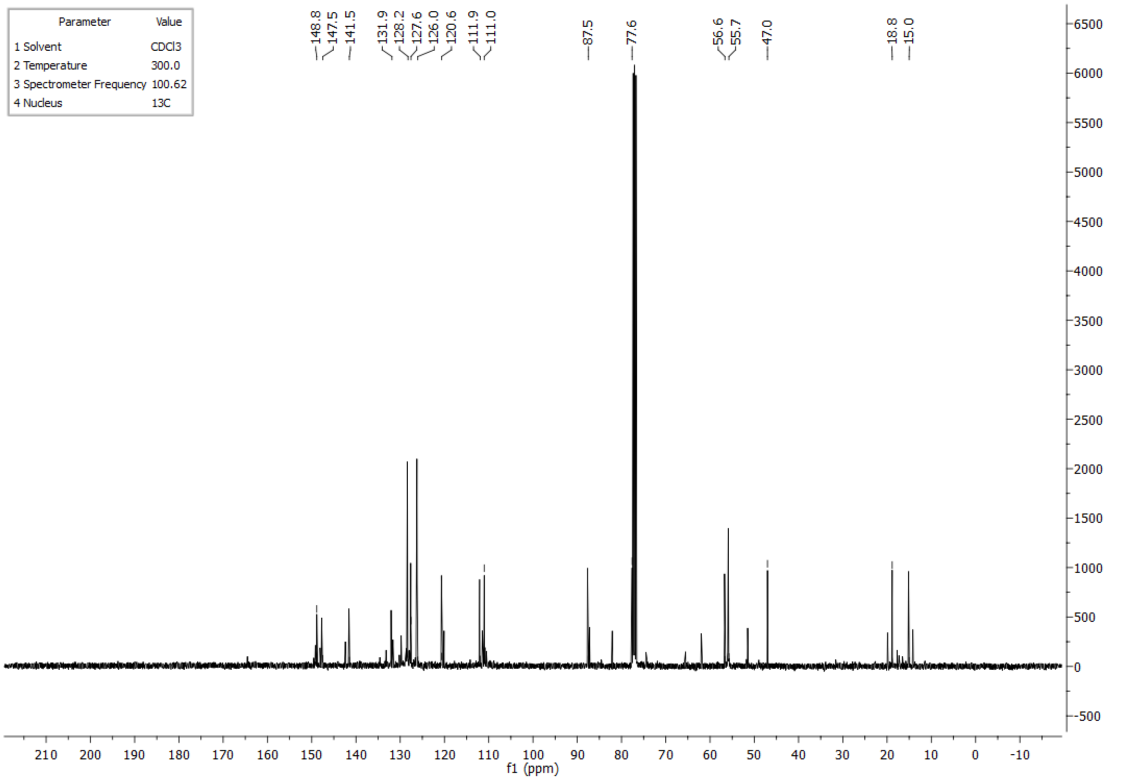
Parameter Value
1 Solvent CDCl3
2 Temperature 300.0
3 Spectrometer Frequency 100.62
4 Nucleus 13C
148.8
147.5
141.5
131.9
128.2
127.6
126.0
120.6
111.9
111.0
87.5
77.6
56.6
55.7
47.0
18.8
15.0
f1 (ppm)

NOESY **1b**

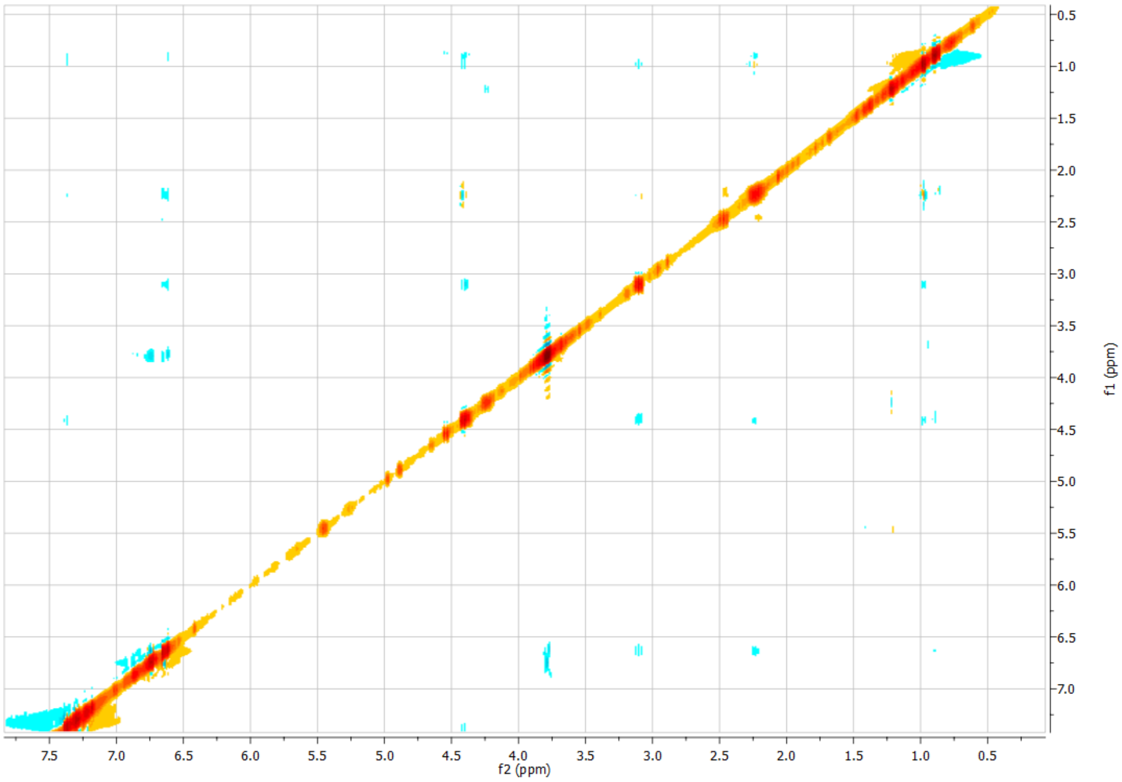

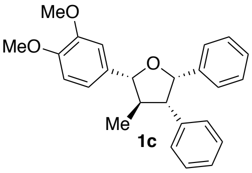
MeO
MeO
O
Me

1c

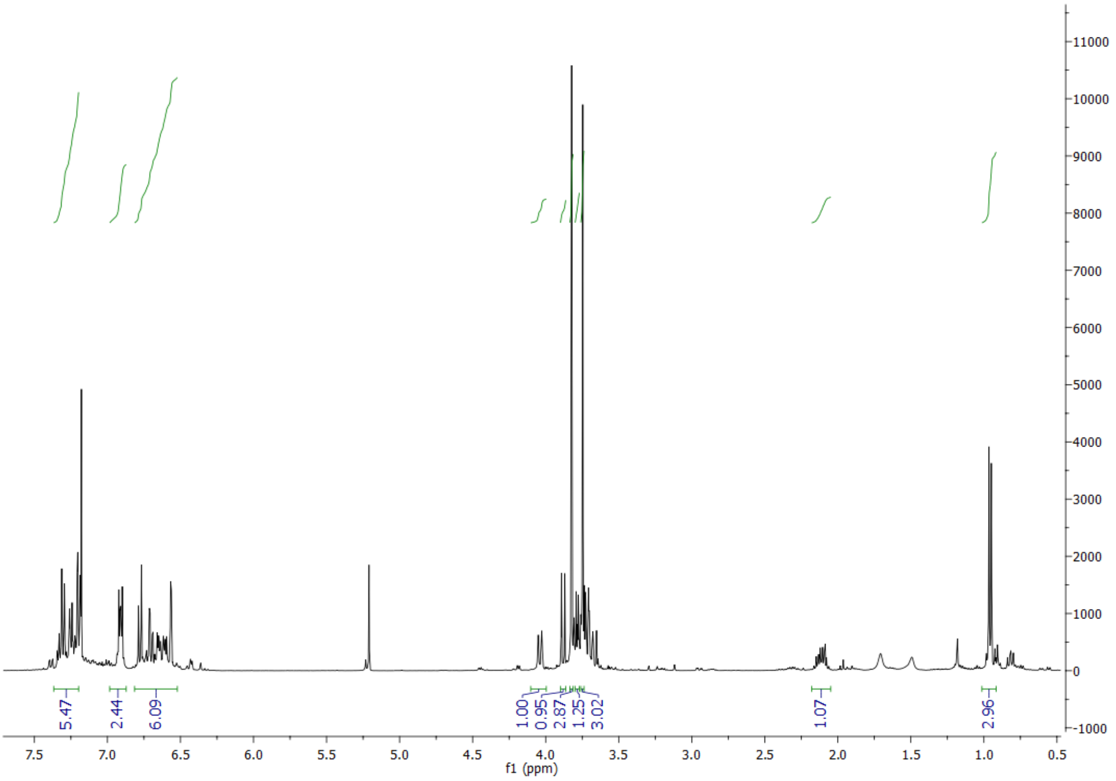
5.47
2.44
6.09
1.00
0.95
2.87
1.25
3.02
1.07
2.96
f1 (ppm)

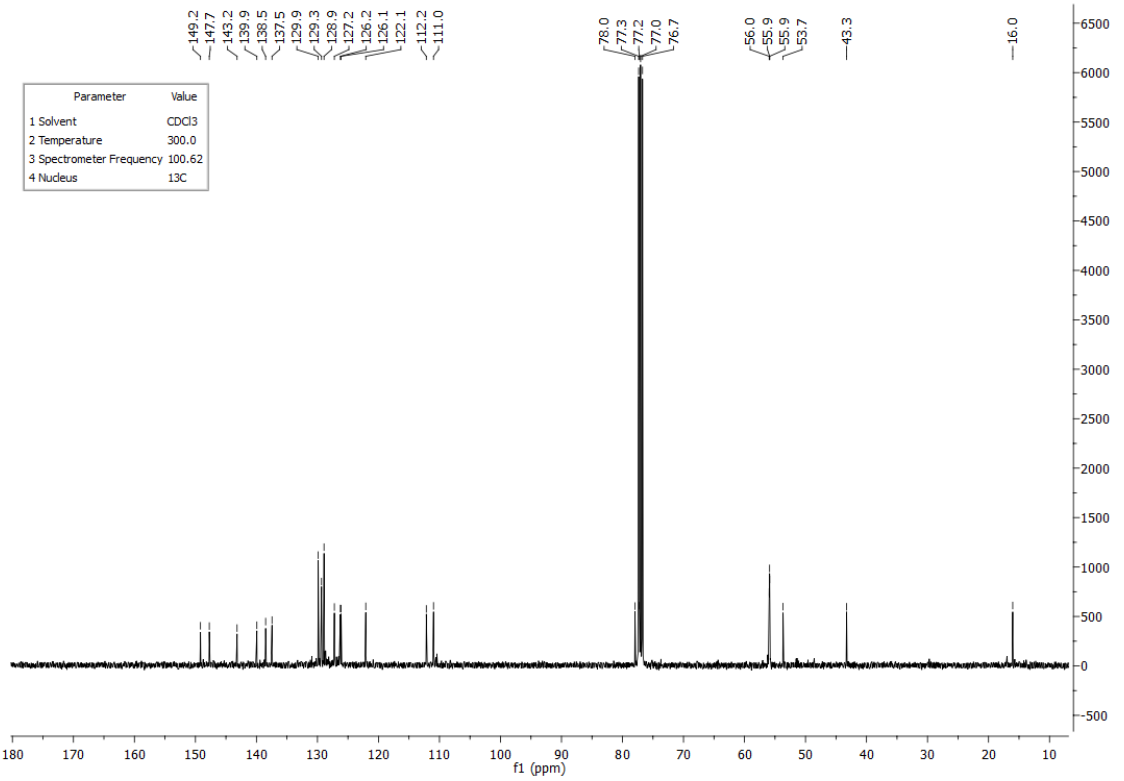
149.2
147.7
143.2
139.9
138.5
137.5
129.9
129.3
128.9
127.2
126.2
126.1
122.1
112.2
111.0
78.0
77.3
77.2
77.0
76.7
56.0
55.9
55.9
53.7
43.3
16.0
Parameter Value
1 Solvent CDCl3
2 Temperature 300.0
3 Spectrometer Frequency 100.62
4 Nucleus 13C
f1 (ppm)

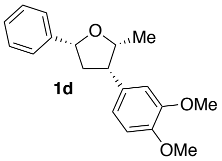

1d

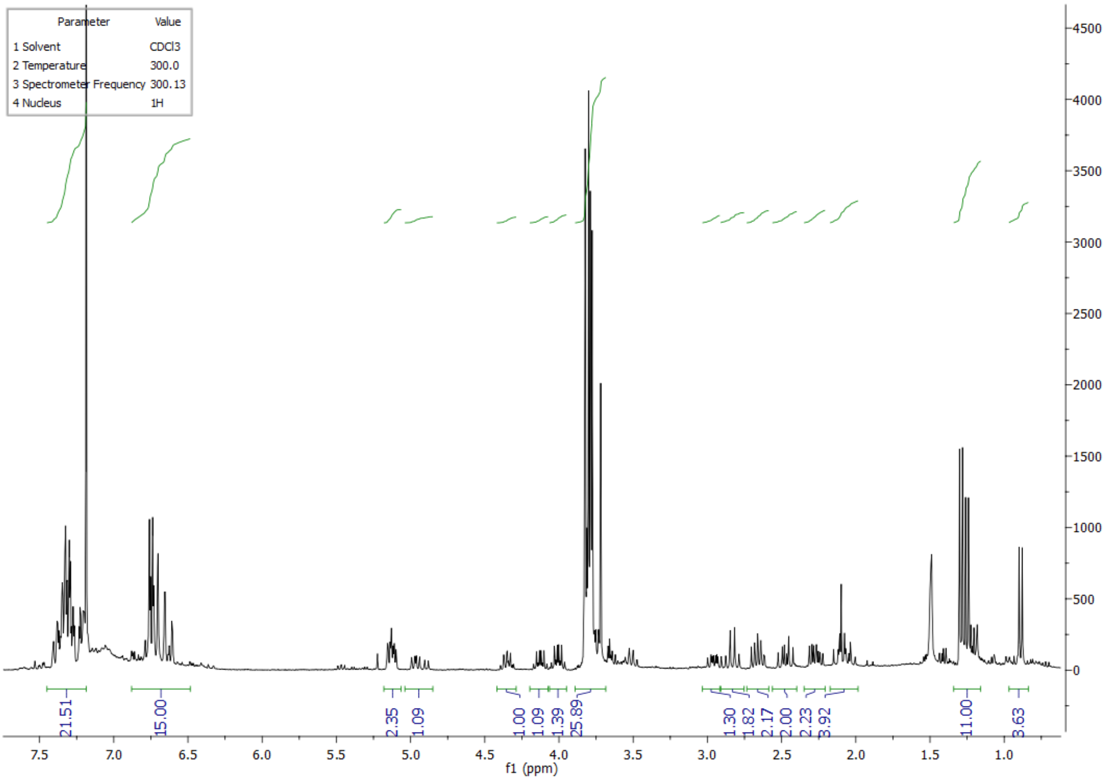

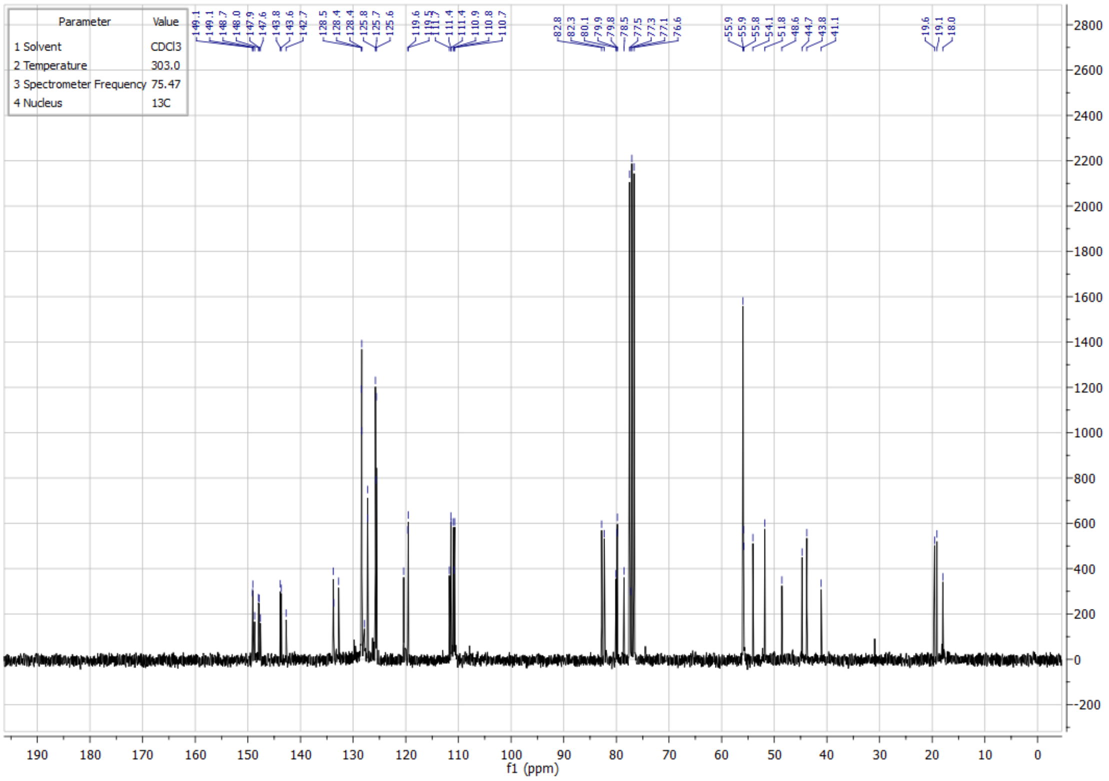

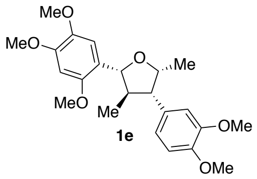
MeO
MeO
MeO
Me
O
Me
OMe
OMe
1e

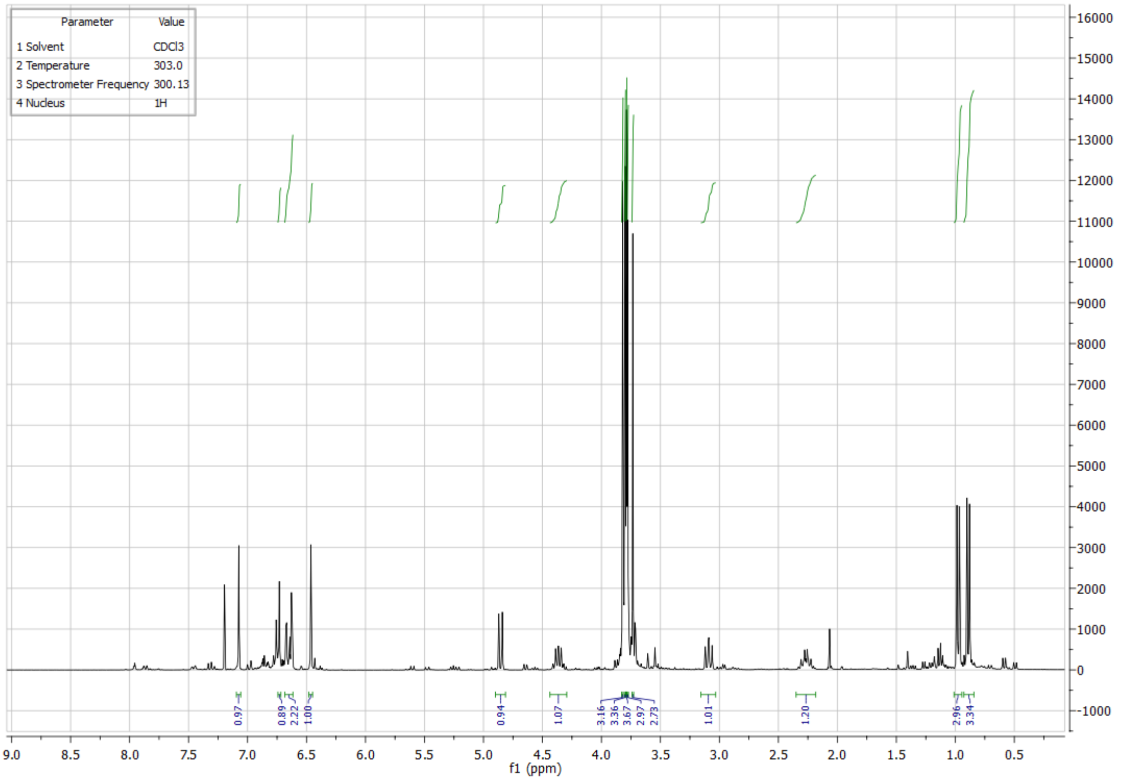
Parameter Value
1 Solvent CDCl3
2 Temperature 303.0
3 Spectrometer Frequency 300.13
4 Nucleus 1H
0.97
0.89
2.22
1.00
0.94
1.07
3.16
3.36
3.67
2.97
2.73
1.01
1.20
2.96
3.34
f1 (ppm)

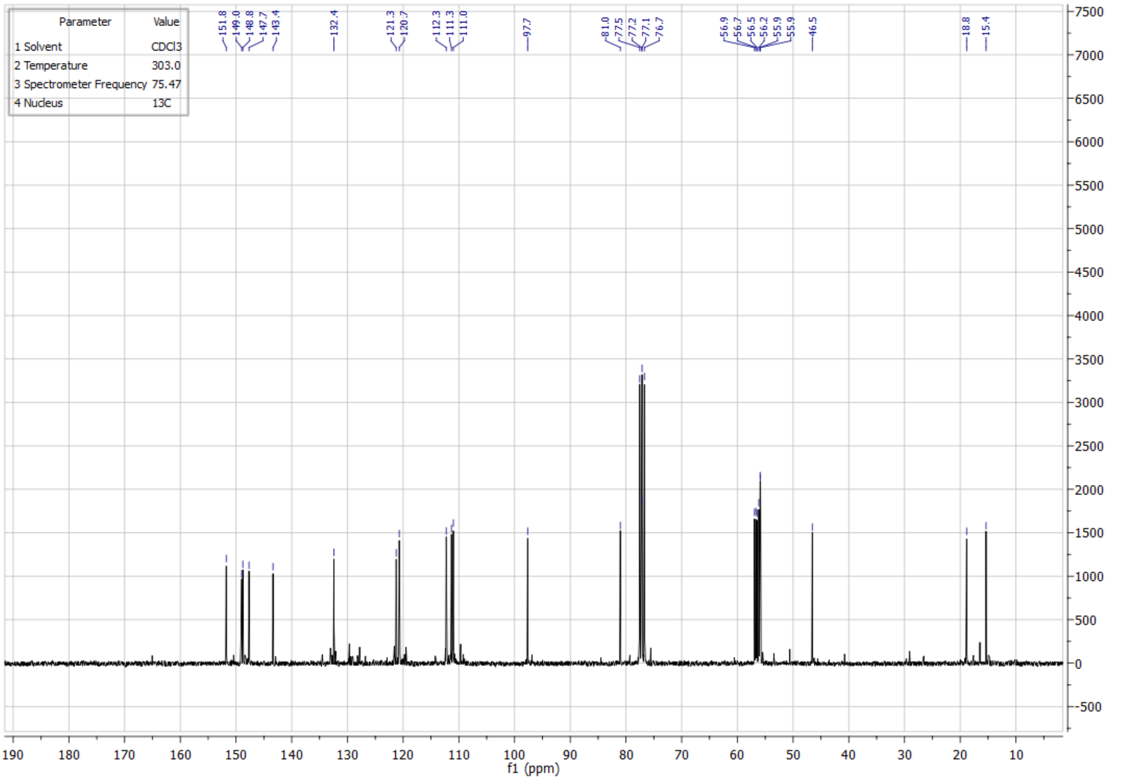
Parameter Value
1 Solvent CDCl3
2 Temperature 303.0
3 Spectrometer Frequency 75.47
4 Nucleus 13C
151.8
149.0
148.8
147.7
143.4
132.4
121.3
120.7
112.3
111.3
111.0
97.7
81.0
77.5
77.2
77.1
76.7
56.9
56.7
56.5
56.2
55.9
46.5
18.8
15.4
f1 (ppm)

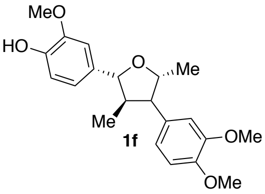
MeO
HO
O
Me
Me
OMe
OMe

1f

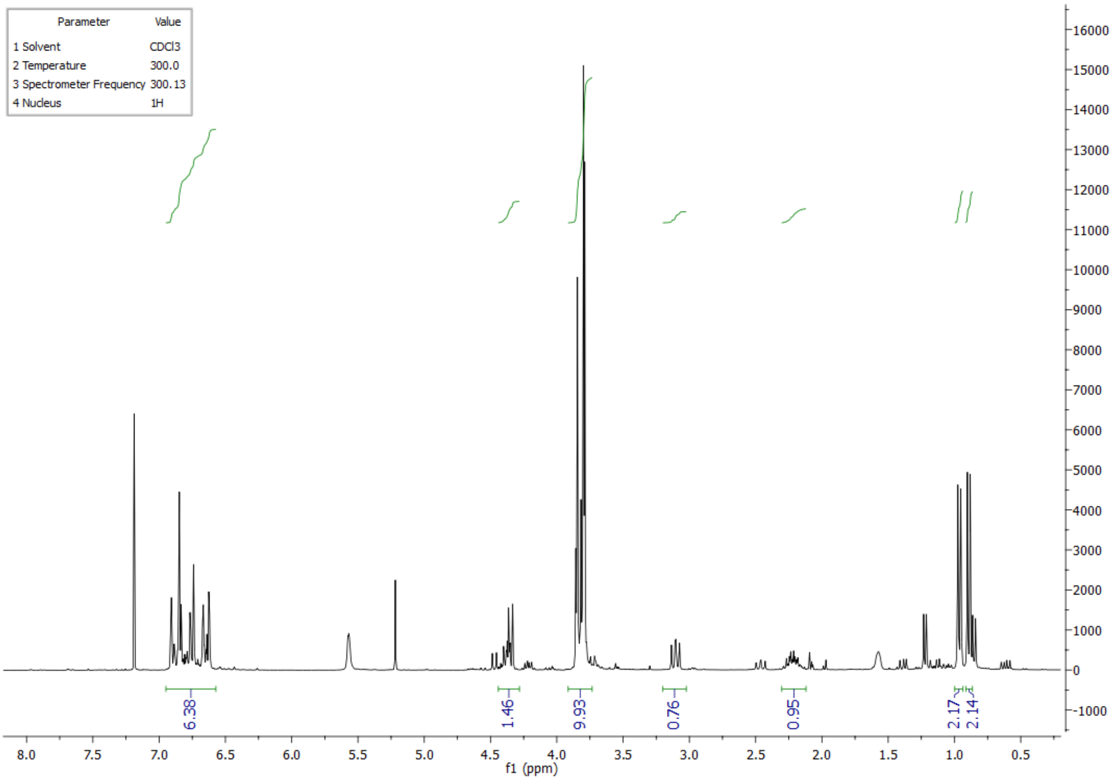
Parameter Value
1 Solvent CDCl3
2 Temperature 300.0
3 Spectrometer Frequency 300.13
4 Nucleus 1H
6.38
1.46
9.93
0.76
0.95
2.17
2.14
f1 (ppm)

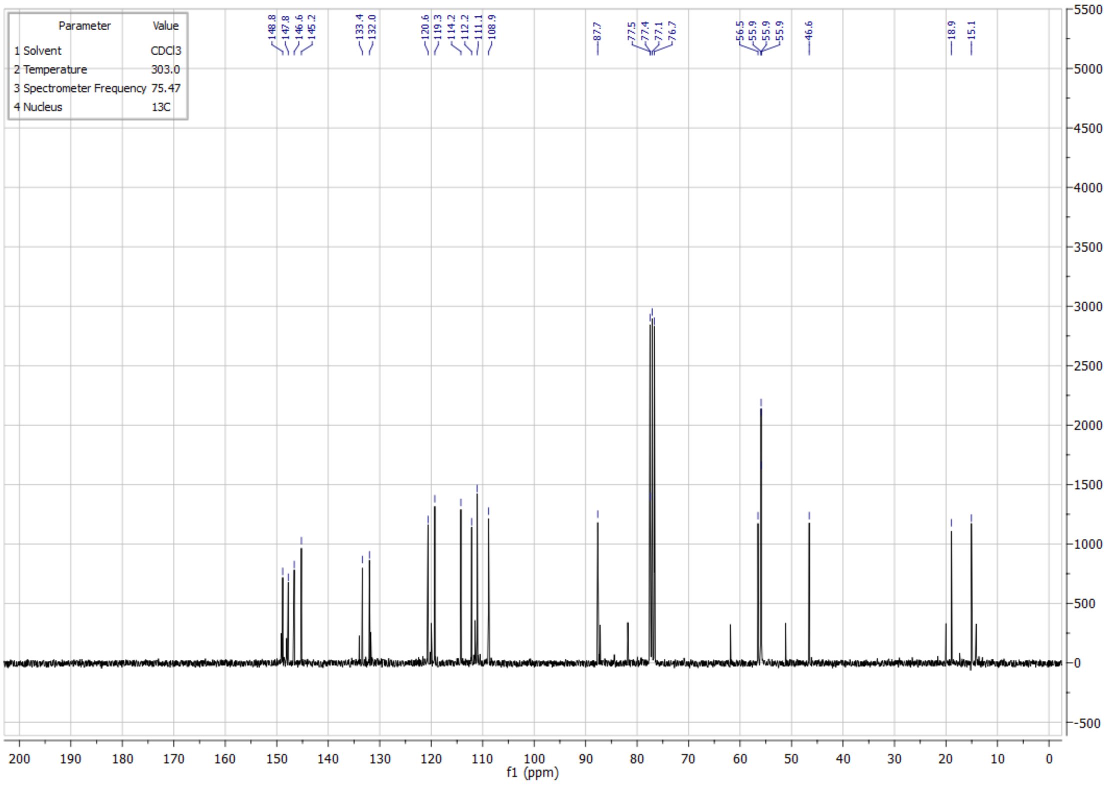
Parameter Value
1 Solvent CDCl3
2 Temperature 303.0
3 Spectrometer Frequency 75.47
4 Nucleus 13C
148.8
147.8
146.6
145.2
133.4
132.0
120.6
119.3
114.2
112.2
111.1
108.9
87.7
77.5
77.4
77.1
76.7
56.5
55.9
55.9
55.9
46.6
18.9
15.1
f1 (ppm)

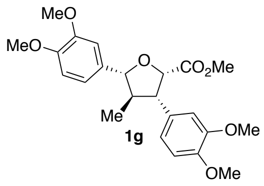
MeO
MeO
O
CO2Me
Me
1g
OMe
OMe

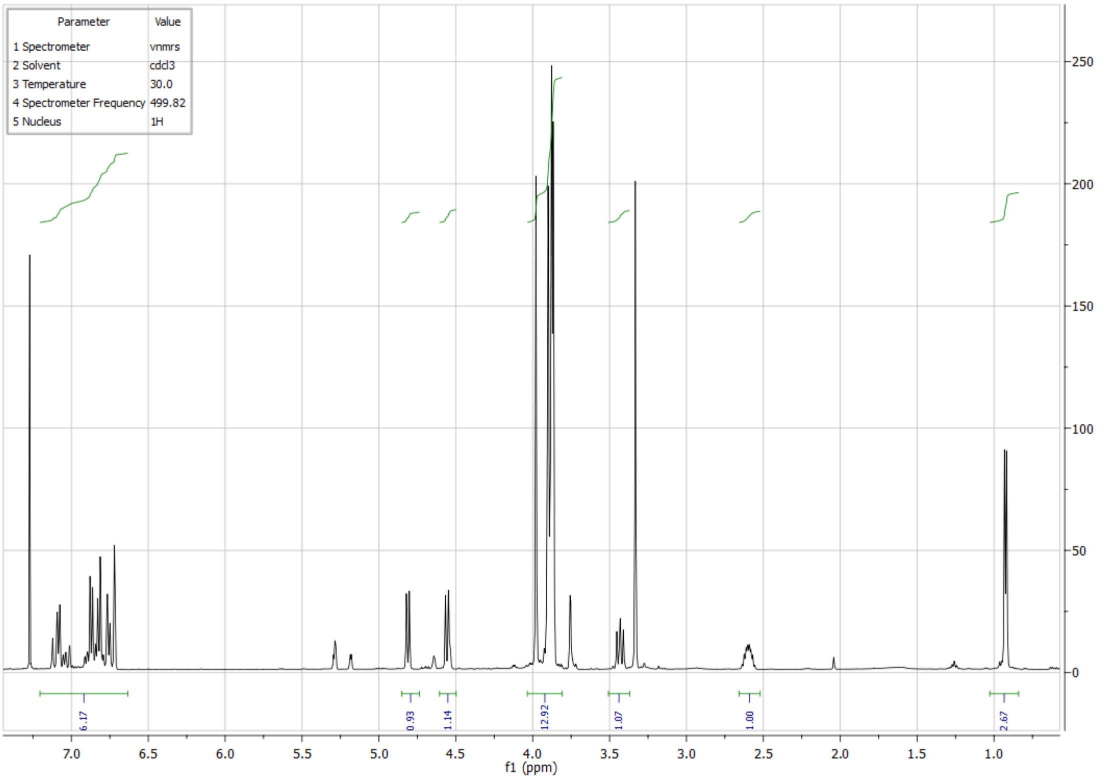
Parameter | Value
1 Spectrometer | vnmrs
2 Solvent | cdcl3
3 Temperature | 30.0
4 Spectrometer Frequency | 499.82
5 Nucleus | 1H
6.17
0.93
1.14
12.92
1.07
1.00
2.67
f1 (ppm)

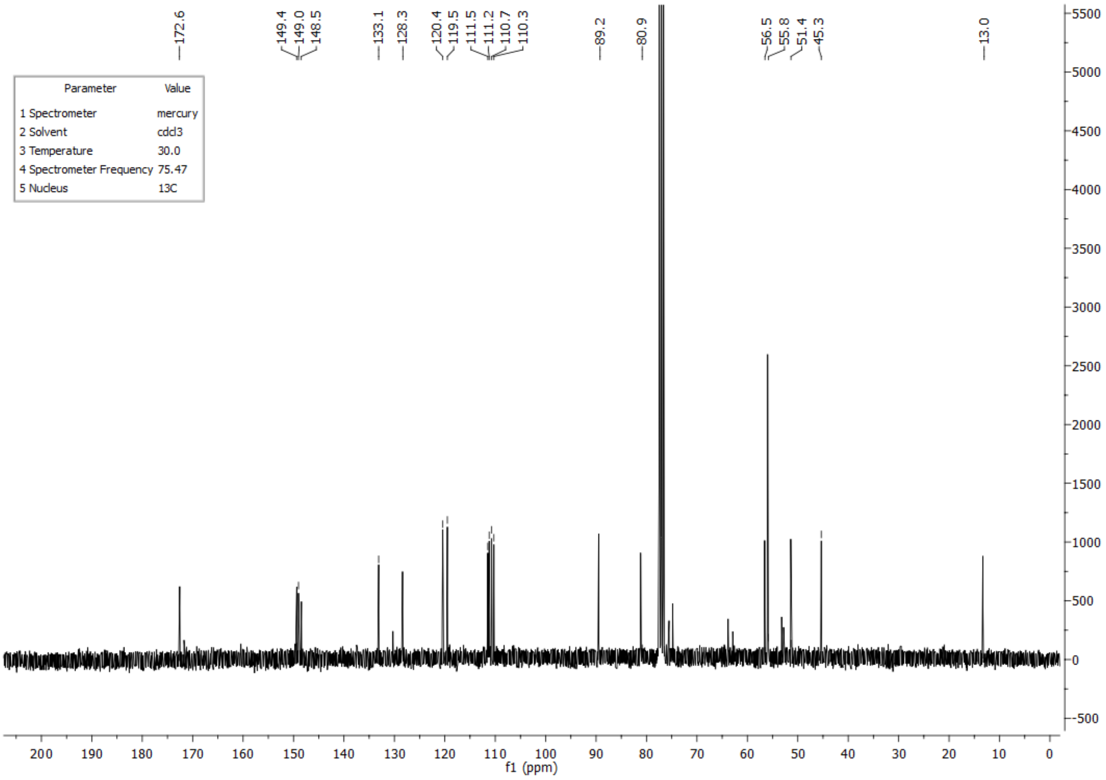
172.6
149.4
149.0
148.5
133.1
128.3
120.4
119.5
111.5
111.2
110.7
110.3
89.2
80.9
56.5
55.8
51.4
45.3
13.0
Parameter | Value
1 Spectrometer | mercury
2 Solvent | cdcl3
3 Temperature | 30.0
4 Spectrometer Frequency | 75.47
5 Nucleus | 13C
f1 (ppm)
